# Supplementary material for: Identification of photoperiod-induced specific miRNAs in the adrenal glands of Sunite sheep (Ovis aries)
Source: Front Vet Sci. 2022 Jul 22;9:888207. doi: 10.3389/fvets.2022.888207 (PMC9354845; doi:10.3389/fvets.2022.888207)
Supplement: Supplementary file 1 [file Data_Sheet_1.ZIP › Supplementary file/miRDeep2 file.zip!/miRDeep2 file/Know_Structure/LP42A2/oar-mir-107.pdf]

miRBase precursor : oar-mir-107
Total read count : 6586
oar-miR-107 read count : 6577
remaining reads : 9

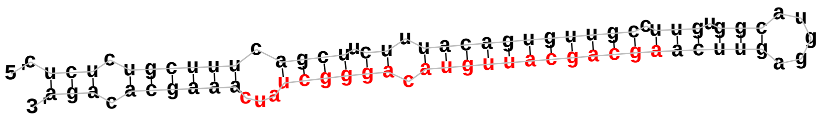

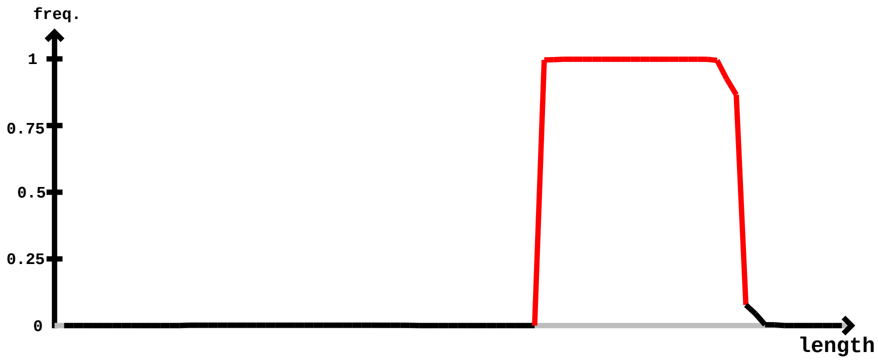

oar-miR-107

```
5'- cucucugcuuucagcuucuuuacaguguugccuuguggcauggaguucaagcagcauuguacagggcuaucaaagcacaga   -3'    exp
    .(((.((((((.((((.((.(((((((((((.(((.(((.....)))))))))))))))))).))))))...)))))).)))     reads      mm          sample
    ............agcuucuuuacaguguugcc.................................................     1          0           seq
    ............agcuucuuuacagugCugccuug..............................................     3          1           seq
    ............agcuucuuuacagugCugccuugu.............................................     4          1           seq
    ............agcuucuuuacaguguugccuugu.............................................     1          0           seq
    .................................................agcagcauuguacaggg...............     2          0           seq
    .................................................agcagcauuguacagggc..............     26         0           seq
    .................................................agcagcauuguacagggcu.............     455        0           seq
    .................................................agcagcauuguacUgggcu.............     1          1           seq
    .................................................agcagcauuguacagggcua............     393        0           seq
    .................................................agcagcauuguacagggcuau...........     5178       0           seq
    .................................................agcagcauuguacagggcuauc..........     217        0           seq
    .................................................agcagcauuguacagggcuauca.........     268        0           seq
    .................................................agcagcauuguacagggcuaucaa........     2          0           seq
    .................................................agcagcauuguacagggcuauGaaa.......     18         1           seq
    ..................................................gcagcauuguacagggcuau...........     5          0           seq
    ..................................................gcagcauuguacagggcuauca.........     1          0           seq
    ...................................................cagcauuguacagggcua............     1          0           seq
    ...................................................cagcauuguacagggcuau...........     7          0           seq
    ...................................................cagcauuguacagggcuauca.........     2          0           seq
    ...................................................cagcauuguacagggcuauGaaa.......     1          1           seq
```
